# Young female migrants and job placement brokers in Addis Ababa, Ethiopia

**DOI:** 10.3389/frph.2024.1241571

**Published:** 2024-01-24

**Authors:** Annabel Erulkar, Eyasu Hailu

**Affiliations:** ^1^ Population Council, International Programs and Social and Behavioral Science Research, New York, NY, United States; ^2^ Population Council, International Programs/Ethiopia Office, Addis Ababa, Ethiopia

**Keywords:** female migrants, migration, brokers, sexual violence, Ethiopia

## Abstract

**Introduction:**

Rates of urbanization in Ethiopia are high and adolescent girls and young women are predominant among those who move from rural to urban areas. Young women frequently undertake rural-urban migration on their own or with a friend, and with little planning for their initial settlement in the city. They frequently rely on job placement brokers to place them into jobs upon arrival, with positions such as domestic work normally including accommodation.

**Methods:**

This is a qualitative study undertaken at the two largest bus stages in Addis Ababa, which are points of arrival for a large number of migrants from rural areas. Three categories of respondents were interviewed in-depth: migrant young women who had arrived within the last few days, job placement brokers who are located in and around the bus station, and market women/vendors at the bus stations who interact with both migrants and brokers.

**Results:**

Migrant girls' point of arrival was an inflection point of risk, especially among girls who were on their own, not accompanied or met at the bus terminal and lacking in plans or preparation of accommodation. Such girls were targeted by thieves at the bus station and by unscrupulous brokers, some of whom forced girls into sexual relations before placing them into paid work. In contrast, market women and some well-meaning brokers took steps to protect girls such as providing temporary accommodation.

**Conclusion:**

This research underscores the need for intensified support to rural-urban migrants to ensure safety and security at the time of arrival at their destination. This includes promotion of pre-migration education and planning; safety assets including sufficient money, cell phones and alternative contacts in the city; and arrangement for immediate, safe and secure accommodation. As a result of the study, a pilot program has been developed, using local resources to extend support for newly arriving migrant girls and young women.

## 1 Introduction

Rates of urbanization in Ethiopia are among the highest in sub-Saharan Africa, with an annual urban population increase of 4.63% (1). Internal, rural-urban migrants in sub-Saharan Africa, including Ethiopia, are increasingly female (2). According to recent research by The World Bank, among rural migrants to Addis Ababa, Ethiopia, 69% were female (3). The same study estimated that rural-urban migrants were aged 22 years, on average, making migrating populations markedly young and female.

Research in Ethiopia—mainly from qualitative studies—has documented the motivations for internal rural-urban migration of young people. Reasons documented include pursuit of schooling or work; to escape rural poverty and lack of opportunities, to escape restrictive gender and social norms and expectations, especially among girls in rural areas; and to exit adverse family situations such as arranged child marriages, following divorce and disputes with family members (4–6). Likewise, these studies have described adolescents' experiences, post-migration, especially in terms of work trajectories and related risks (5–8).

Research comparing adolescent boys' and girls' migration patterns and experiences is limited. One study highlighted how the circumstances of boys' and girls' internal migration differ markedly. Adolescent male migrants from rural areas to Addis Ababa are significantly more likely to move with parent(s) compared with girls (42% of boys and 10% of girls), while girls tend to move with friends, neighbours, distant relatives or on their own. In addition, a significant proportion of girls migrate to escape a forced child marriage in their rural homes (9). With the train network in Ethiopia extremely limited, regional bus travel is the main mode of transport for internal migrants into Addis Ababa (10).

That rural-urban migrant females are frequently runaways from child marriage or make the migration transition outside of a protective family unit, results in a migration transition that is particularly perilous for adolescent girls and young women, exacerbating their vulnerability to sexual abuse and trafficking (11). Indeed, both The World Bank study (3) and the Population Council's monograph, Girls on the Move: Adolescent Girls and Migration in the Developing World (12) highlighted the unique vulnerabilities of migrant girls and young women upon arrival into towns and cities. Temin and colleagues (2013) recommended building mechanisms to “ensure a smooth landing” for migrant girls and young women, including safe accommodation, people to turn to for support and assistance, and building bridges to available networks and services.

Migrant girls and young women often prefer to take domestic worker jobs following migration to towns and cities. This is because the work is familiar and similar to what they routinely do in their rural homes and because most domestic workers in low-income areas of Ethiopia co-reside with their employers, providing them immediate accommodation as soon as they start the job (13). Job placement brokers (known as *delalas* in Amharic) are intermediaries or “go-betweens” who are active in job placement, connecting employers with employees and frequently supplying households, restaurants and bars with domestic workers, waitresses, bar staff as well as commercial sex workers/commercially sexually exploited children. Newly arriving migrants to urban areas of Ethiopia frequently turn to job placement brokers to help place them into jobs, especially when they migrate without a job already in place. Such brokers are frequently stationed at arrival points for migrants—such as at urban bus stages—approaching newly arriving girls to offer job placement, support or advice. Brokers may hold trade licenses and be registered with the Ethiopia Ministry of Labour and Skills, which requires declaring one's business capital and paying taxes on income. Many brokers also work informally, without license or formal registration. Licensed brokers tend to have larger businesses, occupy business premises, and employ other agents/recruiters, whereas unlicensed brokers often work on their own or in informal networks and in open, public locations such as street corners (14).

Recent research among rural-urban migrant girls and young women in Ethiopia reveals that job placement brokers pose both a risk to arriving girls as well as being a mechanism of support (14, 15). The Population Council's research demonstrated that brokers—the vast majority of whom are male—may represent a significant risk to girls, with many taking advantage of their lack of familiarity and naivete to sexually exploit them or traffic them into sex work (14, 16). For example, 44% of brokers in the Ethiopia study reported that they know of other brokers who have sex with their female clients and 37% know of brokers who “trick” or coerce girls into entering sex work/commercial sexual exploitation. Fully 22% of brokers in the study admitted to having sex with their clients, much of which appeared to be nonconsensual and/or coercive (14). This study suggested that unlicensed brokers are more likely to exploit migrants, compared to more well-established licensed brokers who are registered and regulated by government authorities.

While many studies examine the motivations for migration or post-migration experiences, few studies explore the experience of migrants at the point of arrival into destination cities. Rural-urban migration can be unplanned and solitary for many adolescent girls and young women, and they often rely on brokers upon arrival for placement in a job. Using data from different categories of respondents at terminal bus stations, this study examines the experience of migrant girls and young women at the point of arrival into the city, including their preparedness for migration and arrival in the city, their support system and risks they encounter early in the transition to the urban center.

## 2 Methods

This is a qualitative study taking place in two of the largest bus stages in Addis Ababa, Ethiopia, which are arrival points for large number of migrants arriving from rural areas of the country. In these locations, we interviewed three categories of respondents: (1) job placement brokers/employment agents who are located in and around the bus stages and place migrant girls into professions such as domestic work, waitressing and hotel work; (2) market women/petty traders who have small businesses at the bus stage and are familiar with what transpires in those locations; and (3) newly arriving migrant young women aged 18–24. Ultimately, some of these participants were asked to take part in a pilot project aimed to make the arrival of migrants into cities safer, informed and protected.

As a first stage to selecting respondents for interview, we undertook enumeration of employment agents/brokers and market women in both bus stages. Enumeration collected basic information on age, sex, educational level, region of origin, type of work and willingness to be interviewed and/or participate in a project or training. In all 132 brokers and 93 market women were identified in the two bus stages. Out of the 132 brokers identified, 27 refused to be enumerated in the first place, which eliminated them from being selected for interview. Among brokers/agents who gave information for our enumeration, all accepted to be interviewed, in the event they were selected. The initial refusal to be enumerated among brokers/agents is tantamount to a 20% refusal rate (27 out of 132), which is probably due to prevailing sensitivities and stigma related to the profession. Among market women enumerated, 3% refused to be interviewed (3 out of 93). Selection of brokers/agents and market women for interview was drawn from enumeration data and designed to represent the range of ages, places of origin, language groups, years worked at the bus station, and type of work among respondents. Following selection from the enumeration, respondents were approached for interview.

The sampling strategy for newly migrant young women was designed so that respondents are not approached upon arrival to the city, which was assumed to be an extremely vulnerable moment, especially for girls unfamiliar with their new surroundings. Rather, recent migrants were identified and enumerated at agent/broker offices, while waiting for job placement. Eligibility for interview was being a migrant young woman, aged 18–24, having migrated within the last three days intending to settle long term in Addis Ababa, and using an agent/broker for find a job. In all, 81 migrant young women were enumerated, among whom only two (3%) refused to be considered for interview. Following enumeration, young women in brokers’ offices were screen for eligibility, approached for interview and requested to participate in the study. If agreeable for interview, an appointment was made at a convenient time and place for the respondent. In all, 34 broker offices/places of work were used to recruit respondents for the study. Most of the offices belonged to licenses brokers (27), but a few, seven, were the premises of brokers without licenses. It should be noted that brokers with offices—as opposed to those who work on street corners or in other public spaces—are likely to be more well-established and perhaps render more systematic and professional services. The fact that respondents were recruited from the offices of their job-placement brokers may have aided in addressing any reluctance and mistrust on the part of the respondents and resulted in a low number of refusals for interview.

Interview guides were designed for each category of respondent and translated into Amharic. Interviews were carried out face-to-face in the vicinity of the two bus stations, Autobus Terra and Lamberet. All interviews were conducted in Amharic, except for one interview which was conducted in Afan Oromo. In order to compensate for their time, female migrant respondents received a mobile phone recharge card of 50 Ethiopian Birr, which a value of about US $1.

In all, 55 respondents were interviewed including 10 market women, 11 brokers and 34 recent female migrants. Market women and brokers ranged in age from 23 to 50, while migrant girls and young women were aged 18–24.

All interviews were tape-recorded after obtaining informed consent from respondents and permission to tape. In addition, interviewers took detailed notes during the interview. Interview audios were translated and transcribed verbatim, by either the interviewers, themselves, or trained transcribers. Transcriptions were checked alongside the interview audio to ensure accuracy and completeness of the transcript. The research team analysed the transcripts to identify emerging themes and patterns from the data. Illustrative quotes were used to reflect patterns emerging from the data. Preliminary results were shared, discussed and validated in a meeting of the project advisory committee in Ethiopia, which spans a range of governmental, non-governmental and UN agencies working on issues related to migration, labor and gender issues. The study involving human participants was reviewed and approved by both the Population Council and Ethiopian Society of Social Workers, Sociologists and Anthropologists. The participants provided documented informed consent—either written or tape recorded—to participate in this study.

## 3 Results

Most of the young women in the study who were recent migrants had never been to Addis Ababa before and some had not even been outside of their rural home areas. City life was largely new to them and represented a drastic change from their rural homes; many were new to even basic facilities such as electricity and running water, and unaccustomed to crowded urban environments. The migrant girls we interviewed described a varying amount of preparation undertaken before their migration, ranging from long-term financial planning, pre-travel contact with a broker and arranging for accommodation upon arrival, to spur of the moment decision making with little planning, information or plans for accommodation upon arrival. Some of the migrant girls started to plan for their migration to Addis Ababa as much as two years before the actual move. Others had very little preparation or planning, which was particularly common among girls escaping problems in their families, violence or exploitation, forced child marriage, divorce, or conflict.

Addis Ababa was considered by migrants to be a place of opportunity and improved quality of life. While many young women were migrating on their own or with a same-age friend and had little exposure to city life, few described measures taken to ensure their own safety and security, including sexual violence, which was chief among their concerns. Among the young women interviewed, the perceptions of personal safety in the city varied, with some being forewarned to beware of criminals, while others becoming aware of about security concerns only after arriving:

*Interviewer*: Was there something you feared when you came to Addis Ababa?

*Respondent*: Men. I was afraid of men from the time I starting to think of coming to Addis Ababa. I was afraid that I might get raped or some other violence would happen because of them. (Migrant young woman, age 19, 4 years education)

I had no fear [before moving to Addis Ababa]. I decided that things cannot get worse than in my rural home. So, I was not afraid. However, after coming here, I am now scared of the place I am staying. I am constantly worrying about food. (Migrant young women, age 23, 5 years education)

Most arriving migrants brought with them only a small bag of clothes and a small amount of money, or no money at all; some had cell phones and/or telephone numbers of relatives or friends to call if they needed assistance or support. Market women in the study described that some migrant girls and young women arrive with nearly no possessions at all:

They have no money for food. You can ask the petty traders here—there are times when we give them money to buy food. There are also girls who come without clothes with them. They have their bag stolen or lost it on the bus. We get them clothes from the market. A girl can get off the bus with her period. As a woman, you do something to help her. The bus station has positive aspects, but it's a bad neighbourhood because of theft. (Market woman).

### 3.1 Arrival at the bus terminal

Market women and brokers we interviewed had witnessed many migrant girls and young women in distress, and recognized the risk faced especially by girls arriving alone, being new to the city and not familiar with the area. Arriving migrants from rural areas were said to be easily identifiable by people at the bus stages; they are described as usually holding a plastic bag with their belongings and looking confused or overwhelmed, thus making them a prime target for crime, deception or other forms of exploitation:

I often see newly arrived rural girls being robbed and crying. They get lost and don't know where to go. They have language problems—there are those who don't speak Amharic. People from all regions come here. They get robbed and suffer a lot. They can't even do domestic work to earn money to move back home… As a result, they move on to other things and join street life [sex work]. (Market woman)

Some girls arrive when its late and somebody will say, “Let me show you where boarding house is.” Then, they take the girls and steal everything they have and leave them without anything…If you come here after two hours, the area will be filled with gangsters. There are times when I take the girls home when they are coming late, out of pity. (Market woman)

They face a lot of problems. They might get robbed by thieves pretending to be brokers, and they might lie and take them to a hotel and rape them. They face these problems because they do not know anything about the city, and they might get tricked as well… The major problem I see is not having a place to stay. When they have no place to stay, they are in trouble and forced to sleep with men when they don't want to. They have no choice because they have no place to stay. (Unlicensed broker)

Many of the girls we interviewed were met at the bus station by relatives or friends who resided in Addis Ababa, who gave them temporary shelter or helped them find a boarding house/pension in the area until they found a job. This strategy seemed to increase girls' safety upon arrival and provided a source of guidance and advice about the city. While some girls made such arrangements, at times the urban host did not come to the bus stage to receive them, or simply ignored their calls:

*Interviewer:* Have they [friends] provided any support to you since you arrived?

*Respondent:* No, they have not. In fact, they switched off their phones once I got here. (Migrant young woman, age 23, 5 years education)

There was a girl who came to Addis Ababa. She had brothers in the city. The girl thought she would be met by her brothers at the station… We advised her to rent a bed and spend the night nearby, but she refused. I wasn't married at that time, and I lived near St. Michael's Church, so I took her with me for the night. She spent the night crying. She didn't eat anything. She did nothing but cry. When she arrived in the city, she thought her siblings would be waiting for her at the bus terminal.… She had no money or phone with her. She was not too young—she was about 16. (Market woman).

### 3.2 Interaction with brokers

Most migrants in the study had not arranged for a job before travelling to Addis Ababa but had planned to make contact with brokers once they alighted from the bus, most of whom are concentrated around these transportation hubs. Migrants who were met at the bus stage were often referred to a broker through their host friends or relatives. Those who found jobs quickly and had positive experiences with brokers were frequently recommended to specific brokers by family, friends, or contacts in their rural homes:

This broker is not new to us. He helped my sister before. It is my sister who brought me here—she knows him well. He is from our hometown. My sister told me he has brought many girls from our hometown and found them jobs. If it wasn't for him, I wouldn't dare to talk to other brokers. (Migrant young woman, age 19, 12 years education)

Among those who did not have family or friends to receive them, their contact with specific brokers was largely accidental and left to chance. Upon arrival, young women who were not met by friends or relatives at the bus stage, often approached market women and female vendors working at the bus station for advice or direction to a broker. In other cases, market women observed migrants' discomfort or distress and offered their assistance:

I got off the bus and I was wandering around. Then the lady in this shop asked me what I was looking for. I told her that I was looking for a job, and she told me that she can help me find a broker. That is how I met him. (Migrant young woman, age 23, 5 years education)

Most of the migrant young women we interviewed did not know that brokers should be licensed by the government and did not know if the broker they worked with had a license. The migrants we interviewed described fairly positive experience with the brokers, describing them as respectful, helpful and looking out for their best interests:

He is nice. He speaks to us politely. He will not let us go into a house for low pay. He thinks about our wellbeing. (Migrant young woman, age 18, 11 years education)

Given that accommodation upon arrival is lacking for many recent migrants, many of the brokers invested in large, rented rooms where they allowed girls to sleep for one of two nights until they found that placement for them. Girls using these facilities appreciated having a safe place to stay and most brokers did not charge them for the use of the temporary accommodation:

If they don't have a place to stay, we provide that. These are sleeping places for women only. They are not allowed to sleep at the [broker's] office, so if they don't have money for a hostel, we arrange places for them where no men are allowed. (Licensed broker)

We don't give service after 5:30 p.m. to avoid the problems that they might face at night. I also rented a sleeping house for 7000 Birr [equivalent to US $140 per month] for girls in need of accommodation, so they have no problem with where to sleep. (Licensed broker)

Brokers were frequently characterized by market women and brokers alike as being either supportive and ethical or abusive and unethical. In particular licensed brokers view those who are unlicensed as being particularly prone to abuse and exploitation of migrant girls and accuse them of giving the profession of job placement brokerage a bad name. Such brokers were described as raping or sexually coercing migrants, trafficking them into sex work and, generally, taking advantage of their young age, lack of experience in the city and social isolation and lack of support networks. As such, several market women take it upon themselves to ensure migrants' safety and attempt to steer them clear of brokers who might pose a safety risk to them, or give them accommodation in their own homes:

There was one girl who came directly to me and said that she needs work, and she wants to know where the brokers are. But I didn't want her to meet with brokers, so I just talked with other sellers/market women. I asked the women if they know a person who needs a domestic worker, before she goes to the brokers. (Market woman)

Many market women and some brokers described that other brokers sexually exploit girls and require migrants to spend a night or several nights with them before placing them into paid work in the city:

There are some brokers who don't give the girl employment without making her spend the night with him. Some of them are very bad. Especially if there is a girl who he likes, it is a must that he does that. For instance, there was one girl, and the broker told her that the employer can't come today [to take her to the place of work], and that the employer will come the next day…The broker says he will pay for her bed [accommodation] and that she will start work the next day. Then, the broker spends the night with the girl. There are many brokers who make the girls to sleep with them for two or three days. They will never allow girls to start work as soon as they arrive here. They fool them by saying they will find them a job the next day. After the girls do what he wants, he will allow her to start work. This is the behaviour of the brokers. (Market woman)

Girls tell me their stories: “He did this, he made me do that…” Then the girl gets pregnant, but he refuses to accept that the baby is his… When you ask them why they did all these things, they tell you that otherwise the broker would not find them good jobs. The girls say that if they don't sleep with the broker, he won't give them a job. (Market woman)

*Respondent*: There was one girl who came crying after the broker made her sleep with him… she told me that the broker made her sleep with him after she arrived. Then she started crying. She told me that she wants to go back to her rural home. So, she returned because she was very upset…

*Interviewer*: Can you guess her age?

*Respondent*: She can't be more than 15 years old. She was very young… Because she didn't have money, we collected money from the other sellers and security guards at the bus station and helped her to return home. (Market woman)

When interviewers probed respondents about reporting such instances to the police, most said that the police would not follow up the matter or not be helpful in such cases. Some respondents implied that such measures could expose girls to additional sexual abuse.

Despite this, to the reports of sexual abuse by most market women we interviewed, some brokers considered sexual relationships between brokers and migrant young women as largely consensual and, at times, instigated by the girls, themselves:

I don't think migrant girls are touched without them wanting it. You realize she is interested if she goes somewhere with him alone. I don't think brokers rape migrant girls, because times have changed, and no one is raped. Some females are, themselves, interested in having an affair with brokers. I saw this and was surprised. So, I don't think they are raped. (Unlicensed broker)

Such assertions arguably underscore deeply entrenched attitudes that justify and perpetuate the sexual exploitation and harm of girls, especially at a point in their migration journey that is so precarious and fraught with uncertainty.

## 4 Discussion

This study highlighted that the time of arrival into towns and cities is an inflection point for risk among young women migrating from rural areas, especially those migrating on their own, or with little preparation and planning for their first days in the city. Many circumstances combine to significantly heighten a migrant girl's risk during the narrow window of arrival. These include having the appearance of a rural migrant and being a target for thieves; lack of familiarity with urban areas and attendant risks; lack of accommodation upon arrival; language barriers; and lack of protective assets such as accompaniment, money, cell phone and local contacts. The risks are compounded for those who have the misfortune to arrive at sunset or after dark.

Immediate accommodation is critical. Boarding houses and pensions are available but can be expensive and out-of-reach, as well as unsafe places for young women on their own. Unless they have friends or relatives who they can stay with upon arrival, migrants most urgently need to find employment, as jobs, such as domestic work, most often include provision of a place to stay with the employer's family or on the business premises, however modest. Some professional brokers also provide temporary group housing while girls await placement.

Job placement brokers are commonly used by migrant girls and young women to help them find a job. They are critical bridge to settling into urban life, especially among the multitude of migrant girls who lack connections and support in the city. While many brokers provide migrant girls and young with critical support to find work and settle into urban life, some capitalize on their naivete, lack of protective assets and accommodation to abuse and exploit them.

This study has limitations. Migrant girls interviewed in the study were recruited from brokers who had physical offices, with most being licensed by the government. This was done to allay fears and suspicions associated with the researchers and the interview process, and also allow recent arrivals to have identified their strategies for job placement and settlement into the city. However, this recruitment strategy may have biased our sample and account for the relatively positive experiences of migrant girls vis-à-vis their interactions with brokers, compared to the reports of sexual exploitation by market women and brokers in the study. In other words, the challenges faced upon arrival are likely worse than those described by our migrant respondents, because the more isolated and vulnerable young women were less likely to be recruited for the study. Likewise, the reports of exploitation and abuse described by market women and brokers may represent a limited number of individual cases. Regardless, our study findings suggest that there is insufficient support for newly arriving rural-urban migrant girls, especially during the narrow arrival window, when their risk of being targeted for crimes, including theft, violence and sexual abuse, is highest. As Temin and colleagues described (2013), a “smooth landing” is critical. This includes promotion of pre-migration education and planning; safety assets including sufficient money, cell phones and alternative contacts in the city; and arrangement for immediate, safe and secure accommodation. Law enforcement and prosecution of brokers who rape, coerce and exploit migrant girls and young women should be strengthened, and girls and communities should be encouraged to report violations of the law.

Our findings highlight the importance of both pre-departure education and planning, providing young migrants with awareness, skills and assets to address physical and safety challenges upon arrival to a new environment, as well as post-arrival support. The study team and local partners designed and started implementation of a pilot intervention to build support and protection mechanisms for newly arriving migrant girls, using existing structures and resources. Entitled “*Selam Medirashachin”* (Safe Arrival in Amharic), the project recruits and trains market women, brokers, local enforcement, transportation workers and government administrators to promote the safety, security and wellbeing of migrant girls and young women, by providing them with practical guidance for life in the urban area as well as information and referral for social, medical, psychological and legal services. Participants were trained by staff from a local nongovernmental organization active in the bus station. Training sessions included material covering gender and gender-based violence, labor laws, employment practices and contracts, child protection and safeguarding, the circumstances of domestic workers and human trafficking. A qualitative assessment of the pilot project is currently underway. In addition, researchers and local partners are exploring mechanisms for on-arrival education and safety nets, to more fully support the needs of migrant girls and young women, especially those who lack readily available support systems upon arrival.

## Data Availability

The raw data supporting the conclusions of this article will be made available by the authors, without undue reservation.
